# Supplementary material for: C. elegans collectively forms dynamical networks
Source: Nat Commun. 2019 Feb 18;10:683. doi: 10.1038/s41467-019-08537-y (PMC6379388; doi:10.1038/s41467-019-08537-y)
Supplement: Supplementary file 1 — Supplementary Information [file 41467_2019_8537_MOESM1_ESM.pdf]

Supplementary Information

C. elegans collectively forms dynamical networks

Takuma Sugi *et al.*

a b

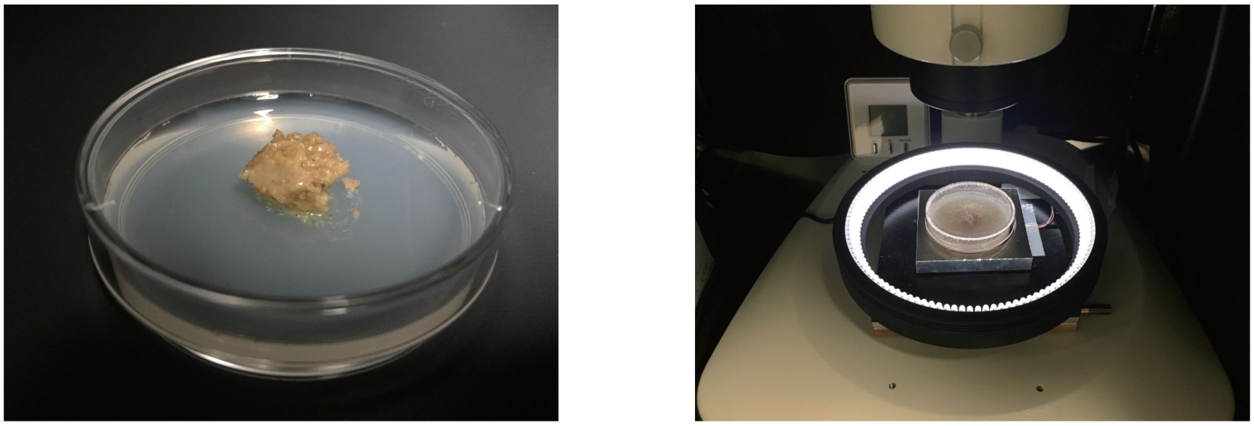

Supplementary Figure 1 Cultivation with DFA in NGM plate. a, DFA was placed on the centre of the agar surface of NGM. The diameter of the Petri plate was approximately 60 mm. **b,** A photo of the experimental system for the observation of *C. elegans* collective motion on a plastic substrate. This system was used for single-worm tracking, optogenetics and humidity change experiments. The temperature of the bottom of a Petri plate inside which worms were propagated on DFA and climbed up onto its lid was controlled by a Peltier temperature control unit on an aluminium plate.

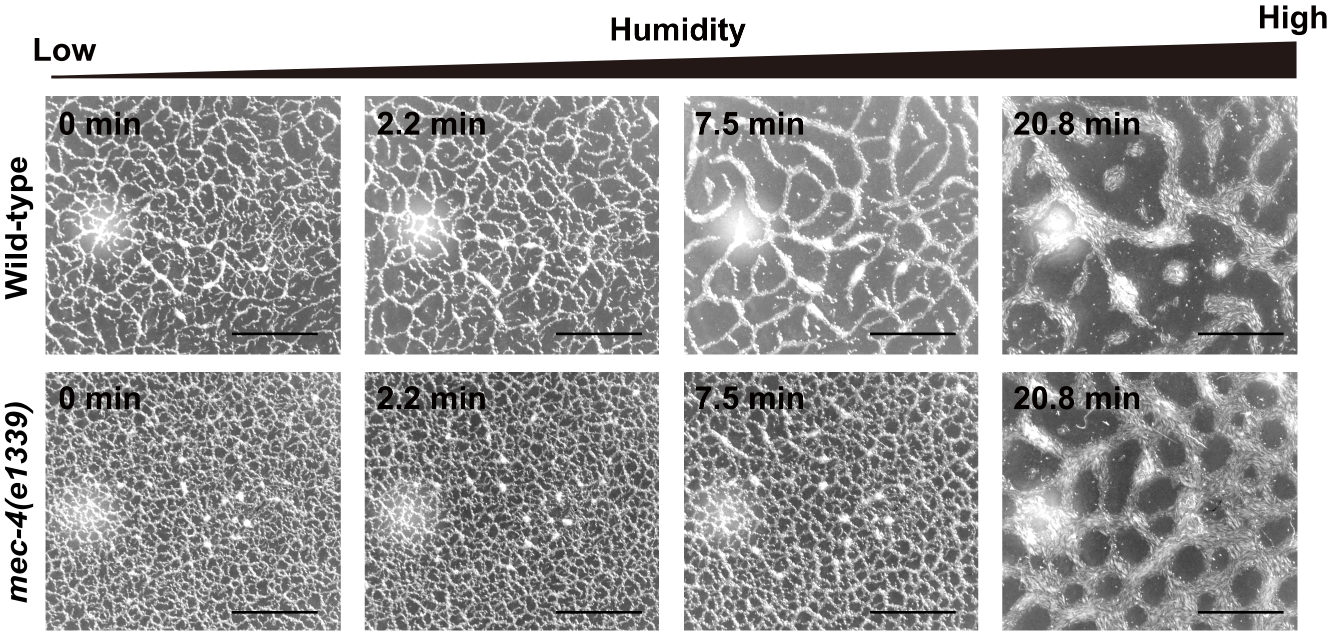

Supplementary Figure 2 Dependence of network pattern on extrinsic parameters. Dependence of the *C. elegans* network on the ambient humidity and movement curvature of isolated single worms. The *mec-4(e1339)* mutant (lower panel) was used as another *mec-4* allele. The average size of the compartments was 0.254 mm^2 at 0 min, 0.334 mm^2 at 2.2 min, and 0.858 mm^2 at 7.5 min in the upper row and was 0.098 mm^2 at 0 min, 0.114 mm^2 at 2.2 min, 0.198 mm^2 at 7.5 min, and 0.541 mm^2 at 20.8 min in the lower row. Scale bar, 4 mm.
