# Supplementary material for: C. elegans collectively forms dynamical networks
Source: Nat Commun. 2019 Feb 18;10:683. doi: 10.1038/s41467-019-08537-y (PMC6379388; doi:10.1038/s41467-019-08537-y)
Supplement: Supplementary file 3 — Description of Additional Supplementary Files [file 41467_2019_8537_MOESM3_ESM.docx]

**Description of Additional Supplementary Files**

**Supplementary Movie 1** | **Dynamical network that emerged on a glass surface.** The

compartment size distribution shown in Fig. 4c was generated from this movie. The movie is

played 500 times faster than the real-time recording rate.

**Supplementary Movie 2** | **Effect of humidity on worm interaction.** Worm behaviours under low and high humidities are shown in left and right panels, respectively. The aligned worms are indicated by magenta circles. attraction and alignment of worms were enhanced by their surrounding water and increased with increasing humidity. This movie was created by cropping Supplementary Movie 3 with contrast enhancement. The movie plays 100 times faster than the real-time recording rate.

**Supplementary Movie 3**| **Dynamical network that emerged on the lid of a Petri plate.**

Wild-type dauer worms were propagated using DFA on NGM in a Petri plate. The worms

self-organized inside the lid. The humidity was changed as described in the Methods section.

Images were taken from above the lid (Supplementary Figure 1). The snapshot shown in the

upper row of Fig. 2b was created from this movie. The movie plays 100 times faster than the

real-time recording rate.

**Supplementary Movie 4​ | ​Running aggregates in higher humidity.​** Images were taken from above the lid of a Petri plate. The humidity was varied as described in the Methods section. The movie is played 300 times faster than the real-time recording rate.

**Supplementary Movie 5​ | ​Dynamical network of ​*mec-4(e1611)​* mutant.​** The ​*mec-4(e1611)* mutants were propagated in the same manner as the wild-type worms (Supplementary Movie 3). The snapshot shown in the lower row of Fig. 2b was created using this movie. The movie plays 100 times faster than the real-time recording rate.

**Supplementary Movie 6​ | ​Optogenetics with blue light exposure for 0.5, 1.0 and 2.0 s and light intensity of approximately 165 µW mm^​−2​^ .​** Illumination is indicated by light blue shading. Short-term illumination (0.5 s) caused no obvious change in worm aggregation. In contrast, at 1.0 s illumination, the worms exhibited a response in which some of the worms temporarily diffused from the aggregates but returned to them. With long-term illumination (2.0 s), the aggregates were disrupted and dispersed. The bundle-shaped aggregate was stable, but the excess activation of the inactive worms caused its collapse. The movie is played 6.7 times faster than the real-time recording rate.

**Supplementary Movie 7​ | ​Optogenetics with blue light exposure for approximately 30 s**. Long-term illumination was performed with mild light intensity (approximately 50 µW mm​^−2^ ). Illumination is indicated by light blue shading. This activation initially caused the arborization and collapse of bundles. Finally, a network different from the initial structure was formed. The movie is played 6.7 times faster than the real-time recording rate.

**Supplementary Movie 8​ | ​Optogenetics with 0.5, 1.0 and 2.0 s blue light illumination without ATR.​** The light intensity was approximately 165 µW mm​^−2^​ . Each illumination is indicated by light blue shading. Note that the illuminations induced no obvious change in the bundles, indicating that the response of the worms was dependent on ChR2 activity. The movie plays 6.7 times faster than the real-time recording rate.

**Supplementary Movie 9**​ | ​**Single-worm tracking.​ Worms exhibit circular movements on a glass surface**. The trajectories indicated in Fig. 3d were created from this movie. The movie is played 500 times faster than the real-time recording rate.

**Supplementary Movie 10​** | ​**Collective motion of the model.​** The average density was 4 per unit length square.​ *k*^a​​^ = 0.002. Initially, **r**​_i​​_ and *θ*​_i​​_ were random. The colour shows the average number density during 10 time units.

**Supplementary Movie 11​** | ​**Collective motion of the model with inactive particles.​** The average numerical density was 4 per unit length square. *k*​^a^​​ = 0.0054. Initially, 30% of particles were inactive. The colour shows the average number density during 10 time units. From 200 to 205 in the left movie (to 400 in the right movie), all particles were active to simulate light stimulation, indicated by light blue shading. The long stimulation led to the collapse of the network.
